# A heterozygous mutation in the *ALPL* gene in an adolescent with Chiari malformation type I accompanied by scoliosis, tethered cord and diastematomyelia

**DOI:** 10.1007/s13760-023-02197-y

**Published:** 2023-01-28

**Authors:** Liqing Xu, Chao Ma, Shengli Shen, Hongzhou Duan, Xiaoyan Li

**Affiliations:** 1 Department of Neurosurgery, Peking University First Hospital, No.8 Xishiku Street, Xicheng District, Beijing, 100034 China; 2 Department of Spine Surgery, Beijing Unicare Hospital, Courtyard 53, South East Fourth Ring Road, Chaoyang District, Beijing, 100023 China; 3 Key Laboratory of Remodeling-Related Cardiovascular Diseases, Capital Medical University, Ministry of Education, Beijing Institute of Heart, Lung and Blood Vessel Diseases, Beijing, People’s Republic of China

Chiari malformation type I (CM-1), scoliosis, tethered cord and diastematomyelia are relatively rare spinal and intraspinal deformities. It is not uncommon for these diseases to occur individually, but it is extremely rare for them to occur simultaneously in a patient. To our knowledge, such a case has not been reported. Herein, we report a case of an adolescent suffering from CM-1 with syringomyelia who simultaneously had scoliosis, tethered cord and diastematomyelia. Whole-exome sequencing (WES) was performed to explore and illustrate the genetic basis.

## Case presentation

A 17-year-old female complained of persistent back pain for 1 month without obvious inducement. It was not accompanied by other symptoms, such as nausea, vomiting, dizziness, numbness, limb weakness or obvious defecation dysfunction. There was nothing special in her past history except scoliosis, which was found around the age of 13 and had worsened in the past 2 years. There was no scoliosis in her family history. Physical examination revealed severe lumbar scoliosis. There were no obvious abnormalities in the blood tests of the patient, and the concentration of alkaline phosphatase was 45 U/L (normal value range: 43–130 U/L).

**Fig. 1:**
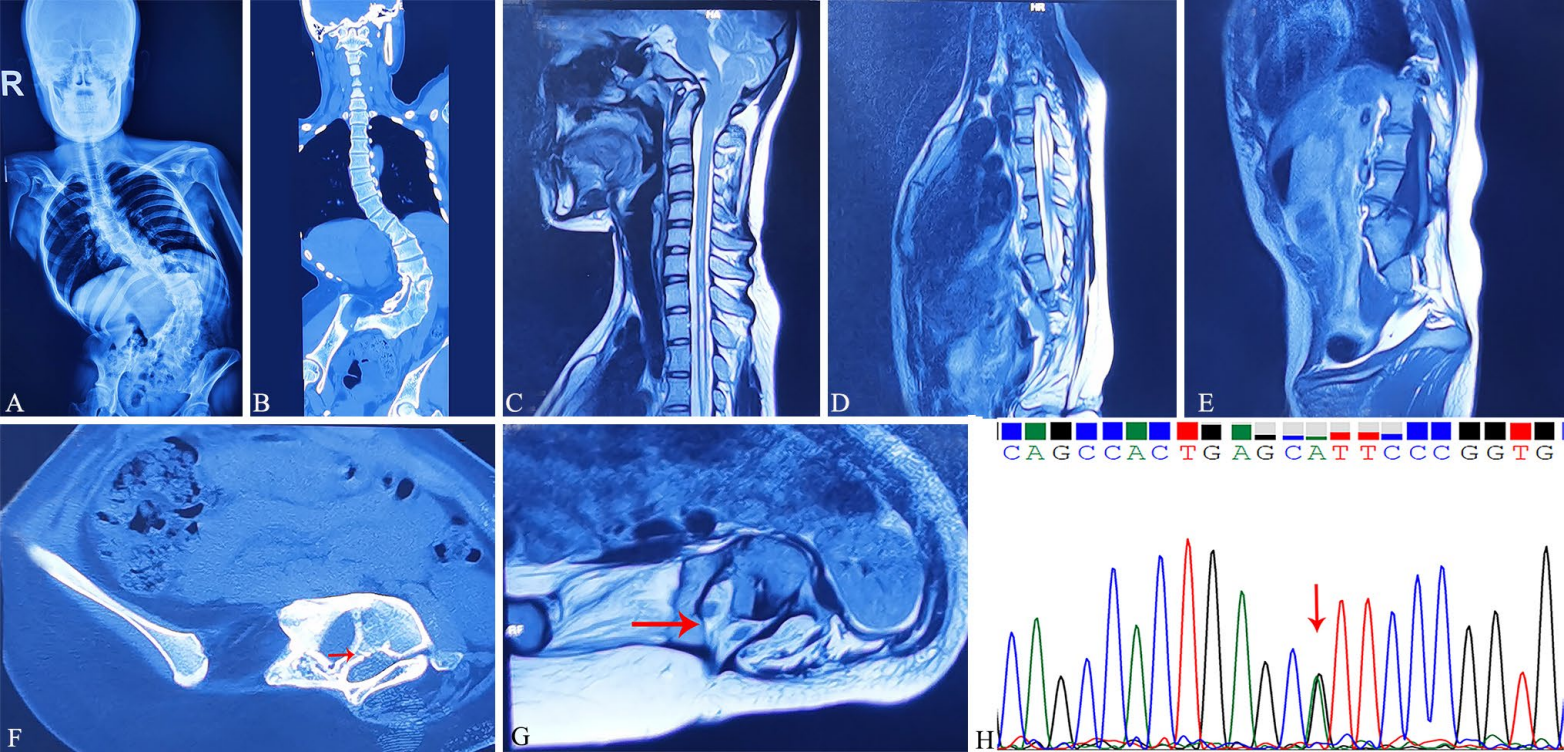
**A** X-ray of the full-length spine showing thoracic and lumbar scoliosis. **B** The coronal image of CT examination showing thoracic and lumbar scoliosis and lower lumbar fusion. **C** The T2 weighted sagittal image of the cervical MRI showing that the cerebellar tonsil herniated downward from the foramen magnum with an enlarged syringomyelia. **D** The T2-weighted sagittal image of the thoracic MRI showing that syringomyelia extended to the T8 level. **E** The T1 weighted sagittal image of the lumbar MRI showing that the end of the conus medullaris was at the L5 level. **F** and **G** The transverse image of CT examination showing a banded bony septum in the L4 spinal canal, indicating diastematomyelia. **H** The sequence chromatogram (red arrow) demonstrating a heterozygous G > A substitution mutation in the *ALPL* gene. (Color figure online)

The X-ray of the full-length spine showed thoracic and lumbar scoliosis (Fig. 1A). CT examination showed L1–L5 lumbar fusion and a bony septum separating the spinal canal of L4 into two cavities, indicating diastematomyelia (Fig. 1B, F, G). The spinal sagittal T2-weighted MRI image showed that the cerebellar tonsil herniated downward from the foramen magnum and obvious continuous syringomyelia from C5 to T8 (Fig. 1C, D). The position of the end of the spinal cord cone was L5, and it was accompanied by thickened and fatty terminal filaments (Fig. 1E).

Based on the above examination results, the patient was diagnosed with Chiari malformation type I accompanied by syringomyelia, scoliosis, tethered cord and diastematomyelia (type 1, according to Peng’s classification). Considering the persistent symptoms and multiple deformities, a single operation to solve all malformations may lead to a long operation time or excessive blood loss. Thus, staged operations were indicated. The first stage included untethering the spinal cord, resection of the bony mediastinum and dural plasty. After the first operation, the symptoms of back pain were significantly improved. Then, 2 weeks later, a secondary surgery for Chiari malformation and syringomyelia was also performed successfully, and the recovery was uneventful. The patient is scheduled to undergo a third operation to correct scoliosis and internal fixation half a year later. There were no complications after the first two operations, and the patient was followed up regularly. Continuous improvement of back pain and no further neurological dysfunctions were observed during the most recent follow-up.

WES was performed to detect the candidate disease-causing variants, and the indicated variant was confirmed by Sanger sequencing. WES identified a germline heterozygous point mutation c. 407 G > A (p.R136H) in exon 5 of the *ALPL* gene (NM_000478, https://www.ncbi.nlm.nih.gov/nucleotide/). The candidate disease-causing variant was verified by Sanger sequencing (Fig. 1H).

## Discussion

CM-1 was previously reported to be accompanied by congenital malformations and resulted in the corresponding symptoms [1, 2]. However, to our knowledge, a patient with concomitant CM-1, syringomyelia, scoliosis, tethered cord, and diastematomyelia has not yet been reported. Therefore, it is worth exploring the underlying mechanisms of such a complex case. We speculate that the formation of a tethered cord is due to the separation and fixation of the spinal cord by the bony septum during its growth [3]. Syringomyelia and scoliosis are secondary to CM-1. The genetic basis of rare CM-1 and tethered cord has been proposed, and the relationship between the two is not simply a traction mechanism [4]. Genetics has played an important role in the understanding of different Chiari malformations, so genetic testing was performed. We found a heterozygous pathogenic mutation of the alkaline phosphatase liver/bone/kidney(*ALPL*) gene by whole-exome sequencing in our case.

The *ALPL* gene is located on chromosome 1p36, and the mutation of this gene leads to hypophosphatasia. Arun et al. reported cases of scoliosis that were associated with hypophosphatasia and described that both were associated with chromosome 1p36 [5]. However, in our case, the patient’s serum concentration of alkaline phosphatase was 45 U/L, which was within the normal range (normal value range: 43–130 U/L). When considering age and sex of the patient [5], we found that this serum alkaline phosphatase level was in the lower limits of the normal range. Hence, we speculate that the series of such rare diseases in this patient may be linked to the *ALPL* gene mutation, and the coexistence has not been described before. Moreover, the heterozygous mutation of the *ALPL* gene might result in a nontypical kind of hypophosphatasia, which may be linked to the series of disorders described here. It may be fascinating to explore their association through more detailed genetic analyses.

## Data Availability

Available if required.

## Abbreviations

CM-1: Chiari malformation type I
WES: Whole-exome sequencing
*ALPL* gene: Alkaline phosphatase liver/bone/kidney gene
